# Mechanical Thrombectomy for Acute Ischemic Stroke After Cardiac Surgery

**DOI:** 10.7759/cureus.1150

**Published:** 2017-04-11

**Authors:** Ali S Haider, Prabhat Garg, Ian T Watson, Dean Leonard, Umair Khan, Ahmed Haque, Phu Nguyen, Kennith F Layton

**Affiliations:** 1 Department of Neurosurgery, Scott and White Hospital, Temple, TX; 2 Texas A&M College of Medicine; 3 School of Medicine, St. Georges University; 4 Department of Anesthesiology, University of Texas Medical Branch at Galveston; 5 Department of Radiology, Baylor University Medical Center

**Keywords:** mechanical thrombectomy, endovascular treatment, ischemic stroke, endovascular neurosurgery, endovascular intervention

## Abstract

Ischemic stroke is a rare yet devastating complication that may occur following cardiothoracic surgery. Fibrinolytic treatment is contraindicated due to elevated risk for hemorrhage. Mechanical thrombectomy entails a catheterized approach wherein the thrombus is physically removed from the vessel without the use of fibrinolytics, minimizing the possibility of intracranial hemorrhage. Here, we present two original cases of mechanical thrombectomy as treatment for patients experiencing emergent large vessel occlusion following cardiothoracic surgery. A literature review was conducted to determine current treatment guidelines, risk factors, and complications resulting from recanalization due to mechanical thrombectomy versus fibrinolytic therapy. One patient was admitted due to chronic, American College of Cardiology/American Heart Association stage D, New York Heart Association functional class IV heart failure and required complete, artificial hemodynamic support for two weeks and on the 19^th^ day experienced neurologic decline secondary to a supraclinoid left internal carotid artery (ICA) occlusion. Mechanical thrombectomy resulted in distal reperfusion and neurologic improvement. The second patient presented with coronary artery disease and underwent triple coronary artery bypass grafting and endovein harvesting. On post-operative day 2, the patient experienced a left ICA occlusion extending to the cavernous ICA resulting in speech impairment and right-sided weakness. The patient was heparinized and underwent mechanical thrombectomy, resulting in immediate speech and muscle strength recovery. Medical advances allow mechanical thrombectomy to be performed in a timely and effective manner at specialized treatment centers. It offers endovascular treatment modalities to a unique patient population with postoperative stroke. In such patients, thrombectomy can safely provide reperfusion while reducing the risk of complications associated with conventional thrombolytics.

## Introduction

Ischemic stroke occurs when an embolus or thrombus occludes a blood vessel supplying nourishment to a portion of the brain parenchyma, resulting in corresponding loss of neurological function. Furthermore, it can occur as a rare yet devastating postoperative complication in patients who recently underwent cardiothoracic surgery [1]. Cardiac procedures such as coronary artery bypass grafting (CABG), valve repair, left ventricular assistance device (LVAD), and certain elecrophysiology (EP) procedures can lead to acute large vessel occlusion postoperatively, with stroke occurring in 2.6% of patients [2]. Though systemic fibrinolysis is contraindicated in these cases due to the increased risk of hemorrhage after a major operation, mechanical thrombectomy has emerged as an effective treatment modality  [3-4]. Here, we present two cases where mechanical thrombectomy was an effective treatment modality for patients experiencing emergent large vessel occlusion (ELVO) after cardiac surgery.

## Case presentation

### Case report 1

A 52-year-old Caucasian male with a history of coronary artery disease (status post right coronary artery (RCA) and circumflex stents) and ischemic cardiomyopathy was admitted to our institution for acute on chronic American College of Cardiology/American Heart Association stage D, New York Heart Association functional class IV heart failure with elevated creatinine and liver enzymes, venous oxygen saturation (SVO2) of 37% and elevated central venous pressure (CVP) of 34 mmHg. On hospital day 2 (HD2), Impella CP (Abiomed, MA, USA) was placed for left ventricular (LV) assist, and dobutamine was added to the outpatient milrinone regimen for inotropic support. The patient underwent ventricular tachycardia with four implantable cardioverter defibrillator (ICD) shocks secondary to hypokalemia. These arrhythmias subsided with potassium repletion and oral amiodarone. On HD4, hemodialysis was started for acute kidney injury (AKI). On HD5, extracorporeal membrane oxygenation (ECMO) was begun. On HD9, the patient was placed on biventricular assistance device (Bi-VAD) support. On HD16, the right ventricular assist device (RVAD) was removed. The patient was hemodynamically stable after the explant. On HD19, the patient experienced an acute neurologic decline. The patient demonstrated global aphasia, right-sided paresis, and had a National Institutes of Health Stroke Score (NIHSS) of 18. A non-contrast head computed tomography (CT) and CT angiogram (CTA) of the head and neck was obtained for initial encounter for stroke-like symptoms. CTA showed occlusion of the supraclinoid left internal carotid artery (ICA) extending into the bifurcation in a carotid "T" configuration with good leptomeningeal collaterals.

**Figure 1:**
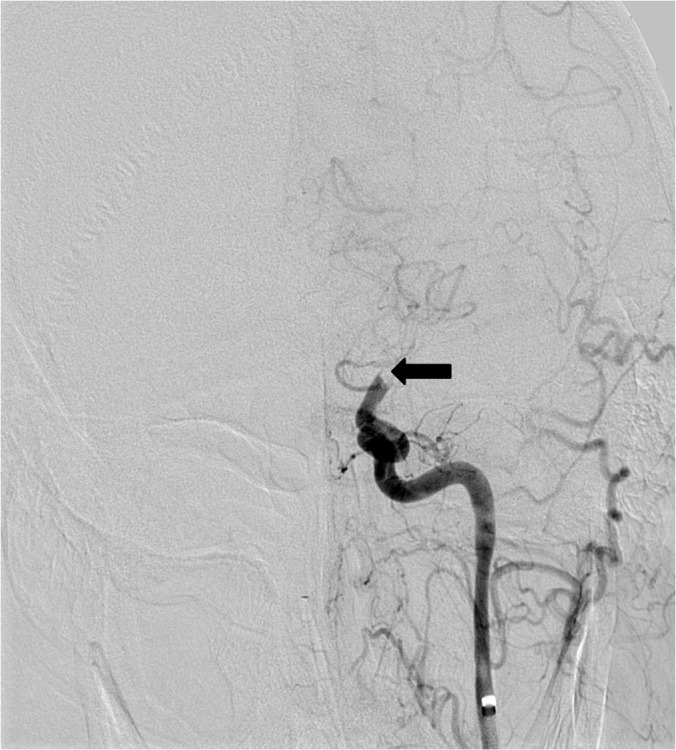
Left internal carotid artery DSA reveals abrupt occlusion of the supraclinoid ICA (arrow) with no perfusion of the anterior or middle cerebral arteries. DSA - digital subtraction angiography;  ICA - internal carotid artery.

**Figure 2:**
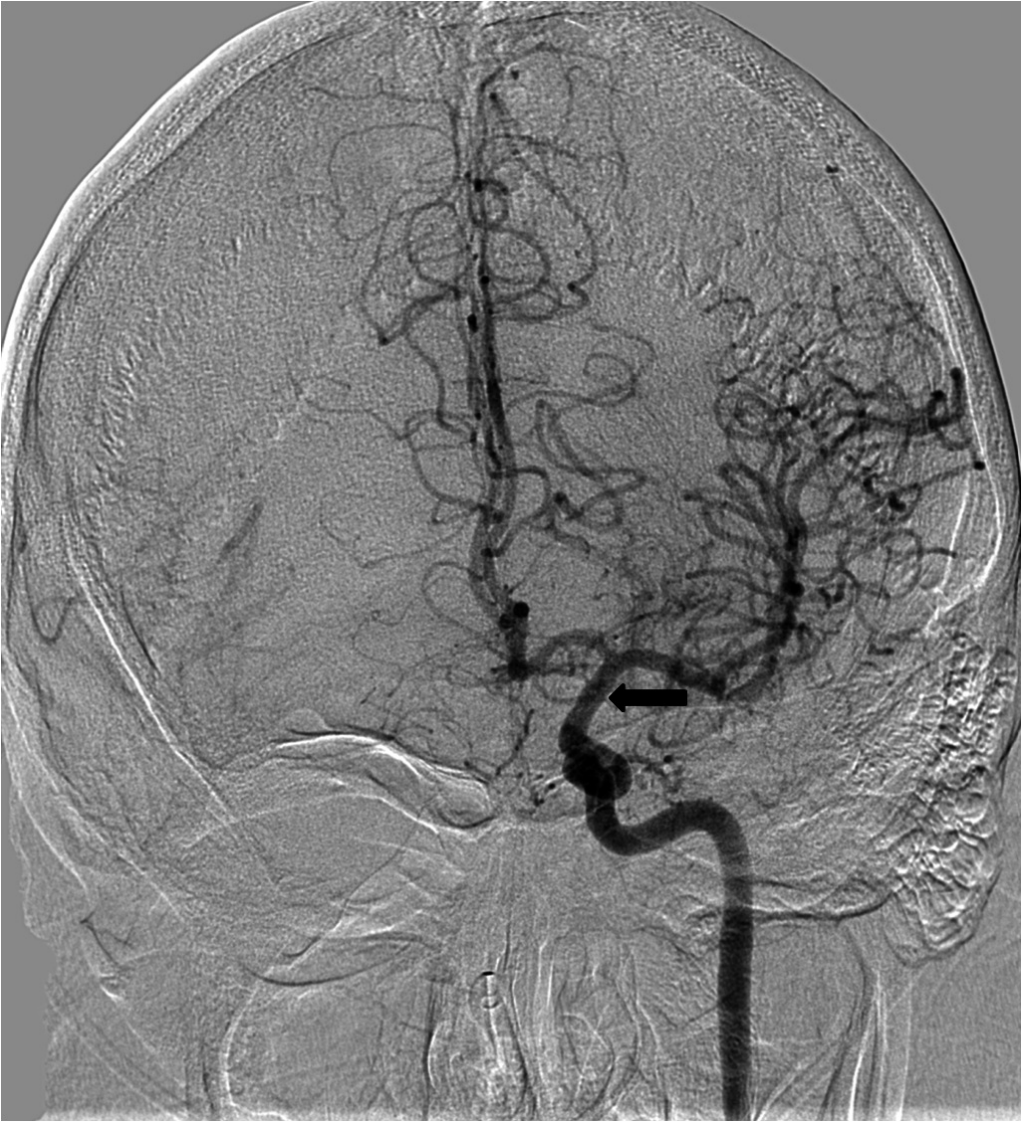
Following mechanical thrombectomy, there is excellent recanalization of the internal carotid (arrow) and left anterior and middle cerebral arteries.

Interventional Neuroradiology was contacted after approximately one hour and 40 minutes from symptom onset. The patient was noted to have an indwelling LVAD and was systemically heparinized. Initial digital subtraction angiography (DSA) revealed an occlusion of the left supraclinoid ICA with no orthograde perfusion of the left anterior or middle cerebral arteries (Figure 1). Mechanical thrombectomy using a Solitaire (Medtronic, Minneapolis, MN) stent retriever was performed, and subsequent DSA demonstrated excellent reperfusion of the left middle cerebral artery (MCA) and anterior cerebral artery (ACA) branches with a thrombolysis in cerebral infarction (TICI) score of 2B (Figure 2). The patient had rapid improvement in neurological function. By the conclusion of the procedure, he was able to verbalize, follow commands, and move his right arm and right leg. Neurology follow-up later that day found a new NIHSS of five, and at discharge the patient demonstrated complete language and motor recovery.

### Case report 2

**Figure 3:**
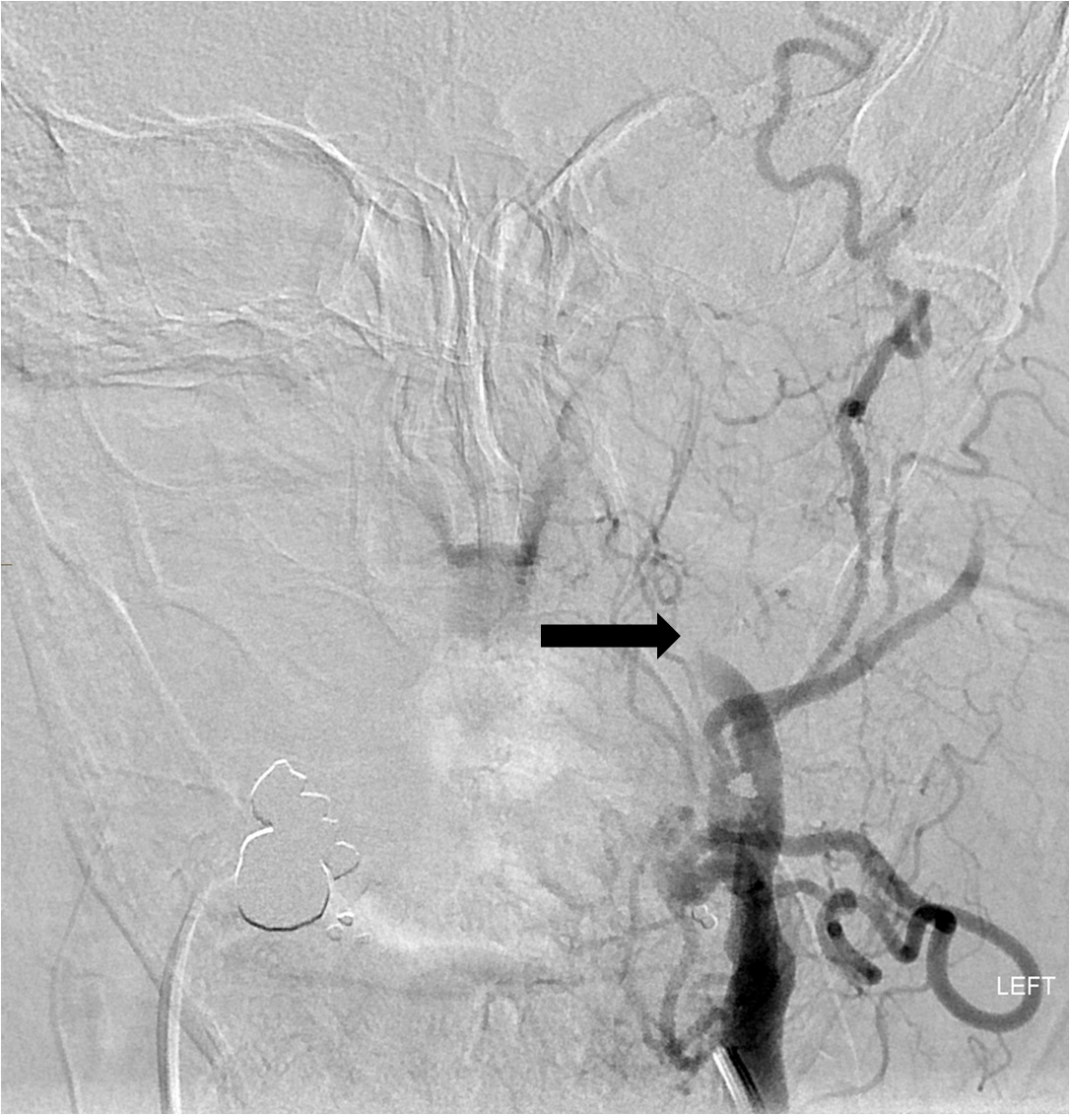
Left common carotid artery DSA reveals stagnant flow in the extracranial left ICA (arrow) related to a complete occlusion of the downstream intracranial ICA. DSA - digital subtraction angiography; ICA - internal carotid artery.

**Figure 4:**
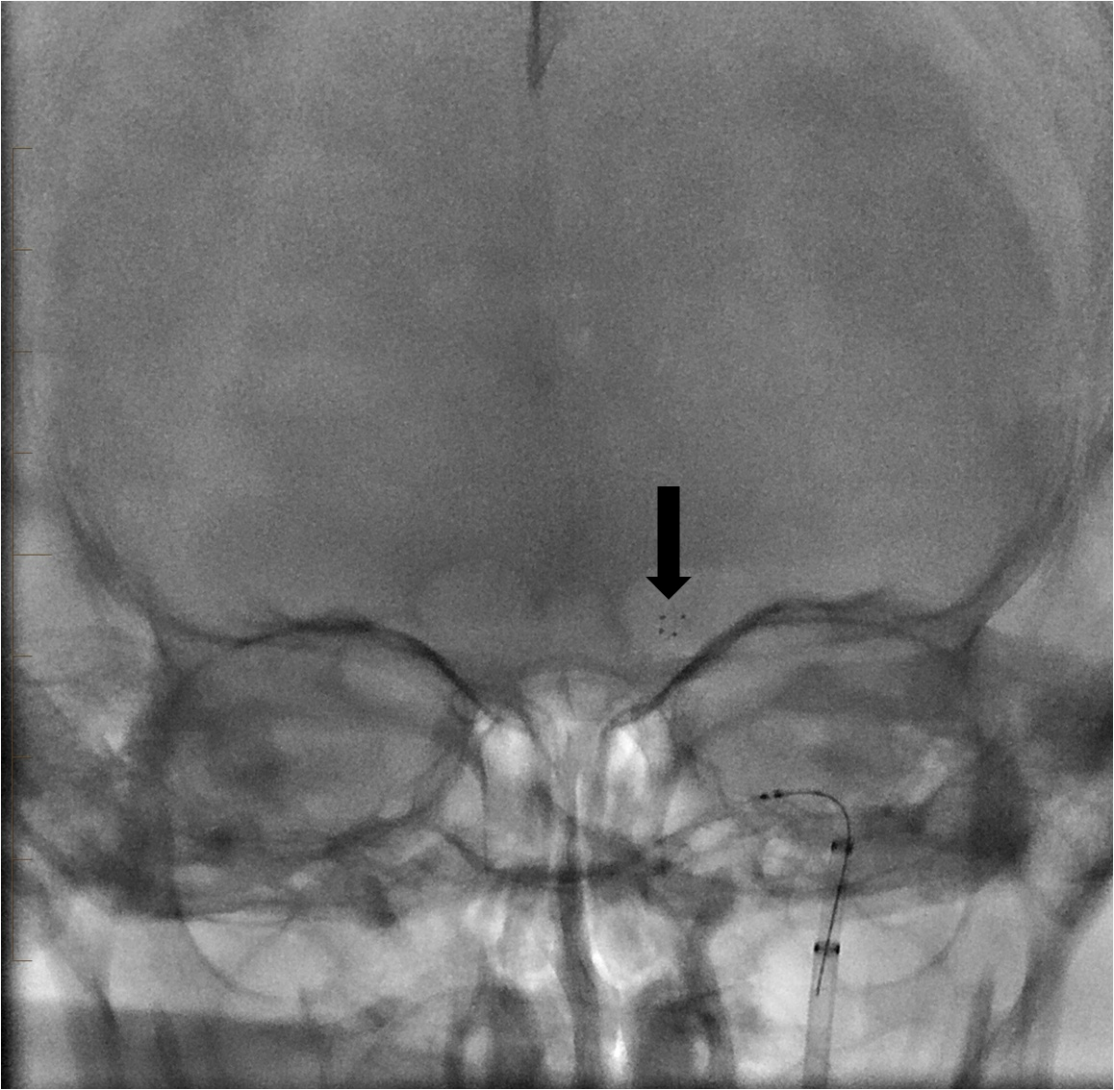
Native fluoroscopic image showing the stent retriever in place with its distal end at the level of the left carotid terminus (arrow).

**Figure 5:**
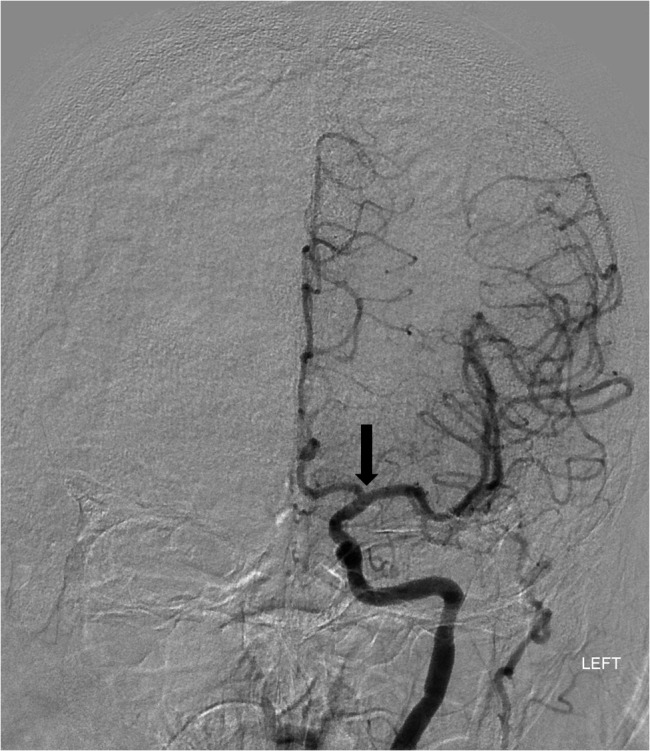
Post thrombectomy left carotid angiogram showing complete recanalization of the left carotid terminus (arrow) and normal flow in the left ACA and MCA branches. ACA - anterior cerebral artery; MCA - middle cerebral artery.

A 75-year-old female with coronary artery disease underwent triple CABG and endovein harvesting. She was admitted to the intensive care unit (ICU) in stable but critical condition. On postoperative day 1 (PD1), she was extubated with systolic blood pressure greater than 90 mmHg and cardiac index greater than 2.2. The following morning, she was noted to have difficulty speaking with right-sided weakness that eventually evolved into aphasia and right-sided flaccid paresis with an NIHSS of 19. Neurology was consulted, intravenous (IV) heparin was started, and neurological imaging studies were ordered. CTA of the head and neck showed an occlusive embolus to the left carotid terminus, extending proximally to the cavernous ICA. The patient was taken to Interventional Neuroradiology for mechanical thrombectomy the same morning. Initial DSA revealed complete occlusion of the intracranial left ICA with stagnant flow in the extracranial ICA due to the distal occlusion (Figure 3). The interventional neuroradiology team successfully removed a 3 cm clot, using a Solitaire (Medtronic, Minneapolis, MN) stent-retriever device, and complete reperfusion of the left ICA was achieved with a TICI score of three (Figures 4-5). Postoperatively, on the angiography table, the patient immediately demonstrated appropriate speech and right-sided movement. Neurology follow-up on the same day also found significant neurologic improvement, as well as a new NIHSS of eight.

## Discussion

The incidence of stroke after cardiac surgery is between 1.7% to 4.6%, with approximately 60% of patients presenting with symptoms immediately after surgery [2, 5-8]. This incidence varies with the procedure: CABG 1.7%, isolated valve 1.8%-3.6%, valve with CABG 3.3%-4.4%, ascending aortic graft 4.6%, congenital 1.0%, and ventricular assist device or transplant 6.2% [2, 5]. The hospital mortality for patients who have had a stroke is substantially higher than for those who have not (32.8% vs 4.9%). In a study that followed 177 patients with ischemic stroke associated with ICA occlusion, 45% of patients died and 75% had either died or were disabled over the course of 420 days, suggesting that ICA occlusion has a very high mortality rate when compared to MCA occlusion alone [9]. Stroke is also associated with substantially longer periods of hospitalization (30 days) versus stroke-free patients (seven days). By the end of the first year there is a 35% absolute survival difference between patients who have not had a stroke and those who have [2].

Several studies have looked at the risk factors and predictors of stroke in patients undergoing cardiac procedures. Positive correlators include age 50-65 (odds ratio [OR] = 2.11), age greater than 65 (OR = 3.22), urgent and emergency surgery (OR = 2.03), aortic valve disease (OR = 2.32), history of atrial fibrillation (OR = 1.88), peripheral artery disease (OR = 1.81), history of cerebrovascular disease (OR = 3.42) and cardiopulmonary bypass time > 110 minutes (OR = 1.71) [8].

Recanalization of emergent large vessel occlusion (ELVO) has been shown to improve outcomes in stroke patients. At three months, functional outcomes are much better in recanalized versus non-recanalized patients with OR = 4.43 (95% CI, 3.32 to 5.91) [4]. Recanalized patients witnessed a decrease in three-month mortality (OR = 0.24; 95% CI, 0.16 to 0.35) [4]. Rates of symptomatic hemorrhagic transformation did not differ between the two groups (OR = 1.11; 95% CI, 0.71 to 1.74) [4]. Recanalization rates were highest in patients who underwent mechanical recanalization as opposed to intravenous or intra-arterial fibrinolytic agents: spontaneous (24.1%), intravenous fibrinolytic (46.2%), intra-arterial fibrinolytic (63.2%), combined intravenous-intra-arterial (67.5%), and mechanical (83.6%) [4]. With new advances in mechanical thrombectomy, revascularization has become more efficient, and mortality has declined significantly. The SOLITAIRE™ with the intention for thrombectomy (SWIFT) trial showed 37% improvement in recanalization with Solitaire stent retrieval compared to older Merci products. Recent thrombectomy trials owe their success to having been performed at high volume, long-standing stroke centers. Additionally, high volume stroke centers, as defined by performing more than 50 procedures annually, have better patient outcomes. Similar successes should not be expected at low volume facilities with less experienced providers and poorer coordination of multispecialty care [10].

## Conclusions

Though stroke patients who have recently undergone a cardiothoracic procedure are poor candidates for intravenous fibrinolysis, interventions can be done to potentially prevent or lessen permanent neurological deficits. With advancements in mechanical thrombectomy, Interventional Neuroradiology can be consulted for timely endovascular recanalization. Literature review revealed that patients experiencing arterial occlusion following cardiothoracic surgery had better outcomes when the vessels could be recanalized. Additionally, recanalization via mechanical thrombectomy had better outcomes than attempts to recanalize with fibrinolytic compounds. This novel procedure offers patients at risk for cerebral infarction a safer alternative therapeutic option where conventional thrombolytics might be too risky to use. If they are not available at the patient’s location, the patient can be quickly transported to a high volume certified comprehensive stroke center for necessary treatment. If not available on-site, it is critical that every facility performing cardiac surgery have a transfer process in place with a well-established, high volume comprehensive stroke center so that time delays to cerebral reperfusion can be avoided.
